# Experimentally designed chemometric models for the assay of toxic adulterants in turmeric powder†

**DOI:** 10.1039/d2ra00697a

**Published:** 2022-03-23

**Authors:** Shymaa S. Soliman, Alaadin E. El-Haddad, Ghada A. Sedik, Mohamed R. Elghobashy, Hala E. Zaazaa, Ahmed S. Saad

**Affiliations:** Analytical Chemistry Department, Faculty of Pharmacy, October 6 University PO Box 12858 6 October City Giza Egypt; Pharmacognosy Department, Faculty of Pharmacy, October 6 University PO Box 12858 6 October City Giza Egypt; Analytical Chemistry Department, Faculty of Pharmacy, Cairo University El-Kasr El-Aini Street Cairo 11562 Egypt ahmed.bayoumy@pharma.cu.edu.eg ahmedss_pharm@yahoo.com; Medicinal Chemistry Department, PharmD Program, Egypt-Japan University of Science and Technology (E-JUST) New Borg El-Arab City Alexandria 21934 Egypt

## Abstract

Turmeric is an indispensable culinary spice in different cultures and a principal component in traditional remedies. Toxic metanil yellow (MY), acid orange 7 (AO) and lead chromate (LCM) are deliberately added to adulterate turmeric powder. This work compares the ability of multivariate chemometric models with those of artificial intelligent networks to enhance the selectivity of spectral data for the rapid assay of these three adulterants in turmeric powder. Using a custom experimental design, we provide a data-driven optimization for the sensitive parameters of the partial least squares model (PLS), artificial neural network (ANN) and genetic algorithm (GA). The optimized models are validated using sets of genuine turmeric samples from five different geographical regions spiked with standard adulterant concentrations. The optimized GA-PLS and GA-ANN models reduce the root mean square error of prediction by 18.4%, 31.1% and 55.3% and 25.0%, 69.9% and 88.4% for MY, AO and LCM, respectively.

## Abstract 2

Turmeric is an indispensable culinary spice in different cultures and a principal component in traditional remedies.

## Graphical abstract

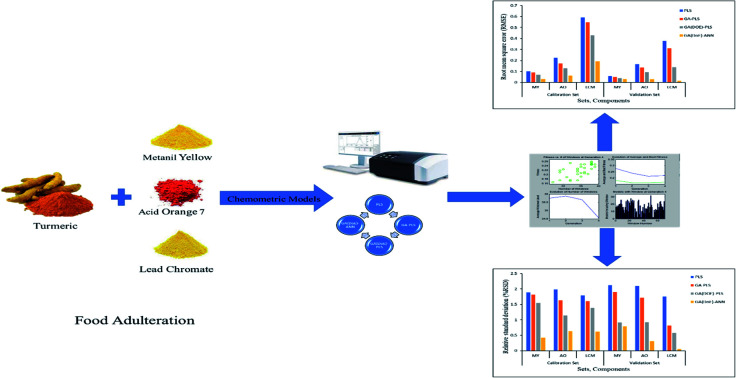

## 1. Introduction

Food provides the human body with the energy necessary to function and exist, provided that safety and quality are guaranteed. The latter is sometimes perturbed deliberately through the inclusion of inferior admixtures or the exclusion of valuable ingredients to increase profit margins at the expense of customer health and social consequences. Laws, traditions and religions incriminate any form of the above practices.

Turmeric rhizome, known as Indian saffron, is one of the largest selling natural food products in the world, and receives a great deal of attention from both medical and culinary specialists.^1^ Turmeric comprises more than 100 compounds. Curcumin is the main active compound and is credited with most of turmeric's health benefits due to its antioxidant and anti-inflammatory properties.^2^ Turmeric can be used to flavor or color many food substances, such as curry powder, mustard, butter and cheese. Medically, it treats several inflammatory conditions,^3^ metabolic syndromes,^4^ inflammatory degenerative eye conditions,^5^ kidney and heart diseases,^3^ cancer,^6^ rheumatoid arthritis,^7^ and several psychiatric disorders.^8^

The relatively high global consumption and demand for turmeric in different applications (such as nutraceuticals, food flavorings and cosmetics) make it more vulnerable to adulteration with low-quality ingredients such as starch, chalk, yellow soapstone, lead chromate and synthetic dyes.^9^ The mentioned adulterants can result in cardiovascular, neurological, hepatotoxic and nephrotoxic health hazards for consumers. For instance, lead chromate (LCM) and synthetic dyes such as metanil yellow (MY) and acid orange 7 (AO) are deliberately added to mimic the color appearance of turmeric despite their hazardous health effects.^9^ The electron-withdrawing character of the azo group in the synthetic dyes sometimes develops an electron deficiency and is reduced to carcinogenic amino compounds.^10^

Long-term consumption of metanil yellow (MY) (sodium 3-[4-anilinophenylazo]benzene sulfonate) causes severe damage to the heart and nervous tissues,^11,12^ degenerative changes in the lining of the stomach, kidneys and liver,^13^ as well as adversely affects the ovaries and testes.^14,15^

Acid orange 7 (AO) (sodium 4-[(2*E*)-2-(2-oxo-naphthalene-1-ylidene)hydrazinyl]benzene sulfonate) and lead chromate are deliberately added to adulterate turmeric powder.^16,17^ Acid orange 7 irritates the eyes, skin, mucous membrane and upper respiratory tract in addition to causing severe headaches, nausea, water-borne diseases, such as dermatitis, and to loss of bone marrow, leading to anemia.^16^ Lead chromate (LCM) can lead to lung and renal cancer, neuropathy, osteopathy, and respiratory tract toxicity.^18^

Analytical chemistry guarantees food safety and quality through the development of reliable sensitive and selective analytical methods to detect and quantify food adulterants and food ingredients.^19–22^ Analytical reports provide authorities, food suppliers and consumers with the evidence necessary to build confidence in food authenticity, safety and quality.^23^

Analyzing pharmaceutical and food products is a highly multivariate process, as many factors (parameters) may be involved during the analysis. Powerful data collection and statistical tools are required to identify the factors that may affect the results of the experiment. Design of experiment technique (DoE) is a robust statistical tool that is successfully deployed in different types of systems, product design, process development and optimization. It plays a prevalent role in decision-making processes through providing useful experimental information, and planning the type and the number of experiments to reach the optimal parameters settings. Consequently, this allows for overcoming the inability defects of generating a large amount of useful experimental data with proper human interpretation.

The DoE technique has been used extensively in several fields, such as chemistry,^24–29^ agriculture,^30,31^ engineering,^32,33^ industry^34,35^ (in particular food industry^36–38^), and even in non-scientific aspects, such as predicting sports results,^39^ especially soccer and basketball. DoE can be used in other disciplines, such as energy^40–44^ and sensors optimization.^45–49^ Previously, many empirical studies in science materials have been made through one factor at a time experimentation (OFAT), which provides uncorrelated material-based systems. This may be attributed to the complexity, safety-critical nature of the energy systems, the high number of components, and the processing conditions. However, DoE evaluates the contribution of different factors simultaneously and defines the needed redundancies for meaningful statistical assessment of the outcomes, allowing for the consistent establishment of energy behavioral-based strategies, a good prediction of energy yields, and integral optimization of electrochemical sensors for higher sensitivity and selectivity.

A literature survey revealed several methods to detect and/or quantify the adulteration of turmeric, such as HPLC,^50,51^ HPTLC,^52,53^ voltammetry,^54^ multispectral imaging,^55^ spectrophotometry^56^ and FTIR.^57–60^ However, the reported literature failed to reveal a single analytical method for the simultaneous assay of MY, AO and LCM in turmeric powder.

In the current work, an investigation and comparison were conducted on the ability of (1) traditional chemometric models, (2) artificially intelligent neural networks, (3) genetic algorithm variable selection tool, and finally (4) experimentally designed optimization for discrimination and simultaneous quantitation of toxic adulterants in the complex natural matrix of turmeric powder. Once fed with a single UV-spectrum of the turmeric powder extract, the models decode the UV-absorbances to discriminate and quantify the adulterants within the complex turmeric powder. The ICH validation parameters were computed to ensure the validity of the method. The model represents a simple, direct and fast analytical tool for quality control laboratories to investigate turmeric samples, and support food suppliers and authorities with scientific evidence regarding food safety and quality.

## 2. Materials and methods

### 2.1 Samples and reagents

Standard metanil yellow (purity 98.00%) and acid orange 7 (purity 98.00%) were purchased from Techno Pharmchem (India). Lead chromate standard (purity 98.00%) was purchased from OTTO (India). Genuine turmeric rhizomes (*Curcuma longa* L.) were purchased from the local markets in Egypt: Ragab (RG), Abu-Auf (ABU), Harraz (HZ) and Medrar (MD). In addition, rhizomes were obtained from Danube (DA) in a Saudi Arabian market. They were kindly identified by the Pharmacognosy Department, Faculty of Pharmacy, October 6 University, Cairo, Egypt. The rhizomes were ground separately to obtain the pure turmeric powder.

Methanol, ethanol, acetonitrile, acetone, acetic acid, nitric acid and sodium hydroxide (NaOH) pellets were purchased from El-NASR Pharmaceutical Chemicals Co., (Egypt). Sodium hydroxide solution (0.2 M) was prepared in distilled water and used as a solvent.

### 2.2 Instrumentation

The spectrophotometric measurements were carried out using a Shimadzu UV-visible spectrophotometer dual beam, model UV-1800 with a 1 cm quartz cell supplied with UV-Probe 2.32 software (Shimadzu Scientific Instruments inc., Kyoto, Japan). All chemometric methods were implemented in MATLAB® 8.1.0.604 (R2013a) and the PLS version 2.1 toolbox. Design-Expert® 13.0.1.0 software was used to analyze the results.

### 2.3 Standard solutions

Stock standard solutions (500 μg mL^−1^) of MY, AO and LCM were prepared using NaOH (0.2 M) as a solvent.

### 2.4 Procedure

#### 2.4.1 Spectral characteristics

Zero-order (D^0^) absorption spectra of separate solutions of 10 μg mL^−1^ MY, AO and LCM, and 200 μg mL^−1^ clear supernatant of each turmeric extract were recorded against NaOH solution (0.2 M) as a blank over the wavelength range of 200–800 nm.

#### 2.4.2 Preparation of the calibration and validation sets

Sets of 25 and 5 mixtures were used for the construction of the calibration set and the validation set, respectively. Each mixture contains different concentrations of the three adulterants spiked to pure turmeric powder. Accurate 20 mg weights of pure turmeric powder (from five different sources: RG, ABU, HZ, MD and DA) were transferred separately into falcon tubes (50 mL), and spiked with standard MY (in the range 200, 400, 600, 800 and 1000 μg), standard AO (in the range 800, 1000, 1200, 1400 and 1600 μg) and standard LCM (in the range 2000, 2500, 3000, 3500 and 4000 μg) according to the multilevel multifactor design. The spiked turmeric powder was sonicated with NaOH (0.2 M, 50 mL, for 15 min), filtered, and the clear filtrate was quantitatively transferred into a volumetric flask (100 mL) and completed using the same solvent.

#### 2.4.3 Wavelength range selection

Different wavelength ranges were sought to select the optimum range that achieves higher sensitivity and selectivity of the proposed models to avoid the noisy regions and poorly informative wavelength range.

#### 2.4.4 Construction of the PLS models

Zero-order absorption spectra of the three adulterants (MY, AO, LCM) were recorded in the wavelength range of 200–800 nm using NaOH (0.2 M) as solvent. The wavelengths in the range of 230–570 nm were selected during the analysis, as the three analytes exhibit adequate absorbance within the working concentration ranges. Cross-validation was carried out using the leave-one-out method, and the root mean square error of cross-validation (RMSECV) was computed and used to obtain the optimum number of latent variables.

#### 2.4.5 Variable selection using the genetic algorithm tool

The genetic algorithm (GA) parameters were configured (Table S1†). The GA procedure was repeated several times to select the relevant wavelengths out of the 341 wavelengths in the range (230 nm–570 nm). The selected wavelengths were used to build the GA-PLS model. The model was used to determine the concentration of MY, AO and LCM.

#### 2.4.6 Design of experiment for the optimization of the GA parameters

A two-level (−1, +1) factorial design was followed using center points to optimize the genetic algorithm parameters. Three numeric factors were manipulated, and two of them were GA parameters. The optimized parameters were the maximum number of latent variables (ml) required to build the GA model, the included fitness percentage (fit%), and the number of the latent variables used to build the PLS model (LV) (Table S2†). The design included three levels for ml (3, 8 and 13), fit% (50%, 70% and 90%) and LV (4, 5 and 6). The optimized parameters of the GA were used to construct the PLS model, and the results were analyzed using Design-Expert® software. RMSEP was calculated to assess the predictive ability of the model.

#### 2.4.7 Artificial neural network

Artificial neural network (ANN) is a computing system that mimics how the human brain analyzes and processes data. The optimized GA data were used to construct the artificial neural network GA(DoE)-ANN. The absorbance matrix was reduced from 341 wavelengths to 123 wavelengths for the three adulterants before presenting them into the network to save the modeling time. The absorbances of the selected wavelengths (123 wavelengths) were used as inputs, while the concentration matrix of the three adulterants was used as output for the GA(DoE)-ANN model. The ANN parameters were adjusted using the Plackett–Burman design (Table S3†).

#### 2.4.8 Analysis of the calibration and validation sets

The PLS, GA-PLS, GA(DoE)-PLS and GA(DoE)-ANN models were constructed to determine the concentration of each analyte in the calibration set, the recovery percent, standard deviation, relative standard deviation (% RSD), and root mean square error of calibration (RMSEC). Afterward, the developed models determined the concentration of each adulterant in the validation set mixtures. The recovery percent, standard deviation, % RSD and RMSEP were calculated.

#### 2.4.9 Reproducibility of the models

The reproducibility of the models was tested using different concentrations of the adulterants. Three concentrations of MY (2, 4 and 10 μg mL^−1^), AO (10, 12 and 16 μg mL^−1^) and LCM (20, 25 and 40 μg mL^−1^) were analyzed three times intra-daily and on three successive days. The % RSD of the three adulterants were calculated.

#### 2.4.10 Application

The literature failed to reveal a quantitative method to assay the three adulterants simultaneously in pure turmeric powder. According to ICH recommendations,^61^ the validation was performed through the determination of the adulterant standards spiked to pure turmeric powder samples from different sources. Accurate 20 mg weights of turmeric powder were spiked with standard MY (in the range of 200 and 400 μg), AO (in the range of 800 and 1000 μg) and LCM (in the range of 2000 and 2500 μg). The mixtures were then sonicated with 50 mL NaOH (0.2 M) in a 50 mL falcon tube, and filtered. The filtrate was then quantitatively transferred into a volumetric flask (100 mL), and completed to the volume using NaOH (0.2 M).

## 3. Results and discussion

Detection of food adulteration has become an increasing concern for governments and institutions to guarantee food safety and quality. Therefore, a single, fast and cheap analytical method was developed for the detection of possible turmeric adulterants. The method should be able to assay the hazardous adulterants in different sources of turmeric powder with minimal sample preparation, only extract and measure. Thus, a simple and cheap UV-visible spectrophotometry method was a good choice to apply. The spectral data were analyzed using multivariate chemometric models. Chemometric combined with spectrophotometric techniques to recognize and assay compounds from their combined spectral data.

### 3.1 Spectral characteristics

**Fig. 1:**
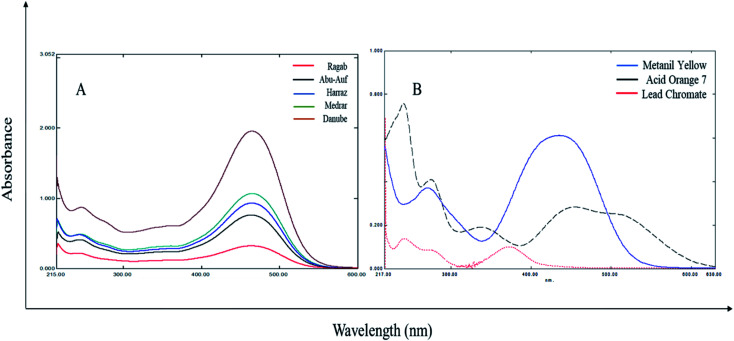
Zero-order absorption spectra of (A) 200 μg mL^−1^ turmeric extracts from different sources, (B) 10 μg mL^−1^ metanil yellow (—), 10 μg mL^−1^ acid orange 7 (- - -) and 10 μg mL^−1^ lead chromate (……).

The absorption spectra of the different turmeric extracts absorb light with a similar pattern, but at different extents. While the absorption spectra of the three adulterants (MY, AO, LCM) show different absorption patterns and extents from each other and turmeric powder (Fig. 1), the application of chemometric was applied for the resolution of the three adulterants in turmeric powder from their spectral data.

### 3.2 Multivariate analysis of different turmeric rhizomes

Five different sources of turmeric rhizomes were purchased from different markets and ground to obtain the pure turmeric powder. Then, the spiked turmeric powders were successfully analyzed using four different multivariate chemometric models, such as partial least square (PLS), genetic algorithm—partial least square (GA-PLS), optimized genetic algorithm—partial least square (GA(DoE)-PLS), and artificial neural network using optimized genetic algorithm dataset (GA(DoE)-ANN), in which each source was given a specific level coded as (−2, −1, 0, 1 and 2).

### 3.3 Solvent selection

Many trials were carried out trying to find the suitable solvent to freely dissolve the three adulterants. Different solvents were tried, such as methanol, ethanol, acetonitrile, acetone, acetic acid, diluted nitric acid and aqueous NaOH. The three adulterants were freely soluble in aqueous NaOH (0.2 M).

### 3.4 Wavelength selection

The wavelengths used were in the range of 230–570 nm, which achieved good linearity for the three adulterants. Meanwhile, the other wavelengths were discarded due to the noise appearing within the range of 200–229 nm and the poor absorbance within the range of 571–800 nm.

### 3.5 Construction of the models

A multilevel multifactor design was used to prepare a calibration set of 25 laboratory prepared mixtures and a validation set of 5 mixtures containing different concentration levels of the three adulterants ranging from 2–10 μg mL^−1^ for MY, 8–16 μg mL^−1^ for AO and 20–40 μg mL^−1^ for LCM. Each model was constructed using the optimum number of latent variables to avoid the unnecessary noise and loss of meaningful data required to build the models.

### 3.6 Implementation of design of experiment technique

The GA reduced the RMSEP, and the standard deviation of the results was obtained using the PLS model. The quality-by-design principles was applied to maximize the accuracy and precision using an experimental design to optimize the parameters of the genetic algorithm-PLS model (ml, fit% and LV). The latter reduced the error and increased the precision of the PLS model for the prediction of MY, AO, and LCM in the five commercially available sources of turmeric. Finally, the resolution of the classical PLS chemometric model was compared to an artificially intelligent neural network. The latter exhibited better predictability for the three adulterants in the five commercial sources of turmeric powder.

### 3.7 Predictive powers of PLS and GA-PLS models

A PLS model was constructed using six latent variables (Fig. S1†). The model successfully determined MY, AO and LCM in spiked turmeric samples within the calibration and validation sets. The mean recovery, % RSD, RMSE and other statistical parameters were calculated for each adulterant (Table 1).

**Table 1:** Assay validation sheet of the chemometric models for the calibration and validation sets

Parameters		Calibration set			Validation set		
		Metanil yellow	Acid orange 7	Lead chromate	Metanil yellow	Acid orange 7	Lead chromate
PLS model^d^	Concentration range (μg mL^−1^)	2–10	8–16	20–40	2–10	8–16	20–40
	Mean	100.09	100.51	100.05	99.00	100.03	99.36
	% RSD	1.892	1.984	1.794	2.120	2.097	1.756
	Repeatability precision (±RSD)	±1.903	±1.924	±1.536	—	—	—
	Intermediate precision (±RSD)	±1.917	±2.084	±1.969	—	—	—
	RMSE	0.101^a^	0.224^a^	0.592^a^	0.057^b^	0.166^b^	0.377^b^
	Slope^c^	0.9996	0.9943	0.9920	1.0025	0.8327	0.9884
	Intercept	−0.0019	0.1163	0.2327	−0.0378	1.5232	0.1192
	*r*	0.9993	0.9970	0.9965	0.9987	0.9998	0.9900
	Latent variables	6					
GA-PLS model^e^	Concentration range (μg mL^−1^)	2–10	8–16	20–40	2–10	8–16	20–40
	Mean	100.14	100.54	99.97	98.95	100.21	98.82
	% RSD	1.814	1.629	1.604	1.894	1.714	0.813
	Repeatability precision ± RSD	±1.799	±2.066	±1.122	—	—	—
	Intermediate precision ± RSD	±1.887	±2.082	±1.642	—	—	—
	RMSE	0.090^a^	0.171^a^	0.547^a^	0.049^b^	0.135^b^	0.309^b^
	Slope^c^	0.9990	0.9865	0.9976	1.0123	0.8790	1.0020
	Intercept	0.0061	0.2099	0.0531	−0.0663	1.1197	−0.3112
	*r*	0.9995	0.9984	0.9969	0.9992	0.9972	0.9979
	Latent variables	6					
GA(DoE)-PLS model^f^	Concentration range (μg mL^−1^)	2–10	8–16	20–40	2–10	8–16	20–40
	Mean	100.22	100.24	100.05	99.40	99.52	99.58
	% RSD	1.542	1.143	1.388	0.918	0.919	0.576
	Repeatability precision (±% RSD)	±1.540	±1.785	±1.398	—	—	—
	Intermediate precision (±% RSD)	±1.687	±1.859	±1.589	—	—	—
	RMSE	0.071^a^	0.127^a^	0.428^a^	0.040^b^	0.093^b^	0.138^b^
	Slope^c^	1.0000	0.9968	0.9878	0.9856	0.9596	1.0166
	Intercept	0.0034	0.0624	0.3587	0.0256	0.3245	−0.4702
	*r*	0.9997	0.9989	0.9982	0.9994	0.9972	0.9993
	Latent variables	5					
GA(DoE)-ANN model^g^	Concentration range (μg mL^−1^)	2–10	8–16	20–40	2–10	8–16	20–40
	Mean	100.02	100.05	100.17	100.15	99.86	100.02
	% RSD	0.415	0.631	0.613	0.782	0.313	0.054
	Repeatability precision ± RSD	±0.564	±0.932	±0.584	—	—	—
	Intermediate precision ± RSD	±0.722	±1.167	±1.052	—	—	—
	RMSE	0.030^a^	0.062^a^	0.191^a^	0.030^b^	0.028^b^	0.016^b^
	Slope^c^	1.0015	0.9974	1.0039	1.0113	0.9879	1.0019
	Intercept	−0.0011	0.0400	−0.0615	−0.0226	0.1016	−0.0343
	*r*	0.9999	0.9997	0.9997	0.9997	0.9997	1.0000
	Latent variables	5					

a Root mean square error of calibration.

b Root mean square error of prediction.

c Data of the straight line plotted between predicted *versus* actual concentrations.

d Partial least square.

e Genetic algorithm-partial least square.

f Optimized genetic algorithm-partial least square.

g Artificial neural network using optimized genetic-algorithm.

The small RMSEP values indicate good predictability and high-resolution power of the model. A good correlation coefficient (*r*) for each adulterant was achieved, which indicates a good fit between the predicted and the actual concentrations.

**Fig. 2:**
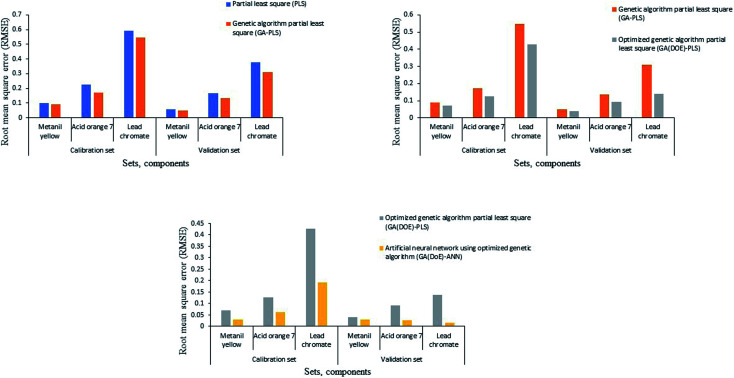
Root mean square error of calibration and validation sets for the three adulterants using the four chemometric models.

Genetic algorithm tool improved the predictive power of the PLS model by selecting the most informative wavelengths and excluding the less informative ones. It reduced the number of wavelengths to about 68.9% of the original ones (106 wavelengths for the three adulterants). We constructed the GA-PLS model using the GA-selected wavelength of the calibration set. RMSECV calculations show that six latent variables are adequate for the construction of the GA-PLS model (Fig. S1†). Unfortunately, GA did not reduce the number of latent variables compared to the previously mentioned PLS model. The RMSEP values were relatively small compared to that of the PLS model (Fig. 2), which indicates an increase in the predictive power of the GA-PLS method compared to the classical PLS method.

### 3.8 Optimization of GA-PLS model

Even with the preference of the GA-PLS model over the classical one, there was a necessity to optimize its predictive ability using the design of experiment technique (DoE). The DoE evaluates the impact of different factors on the desired response, and identifies important interactions that are missed when experimenting with one factor at a time. The results were analyzed using one-way ANOVA (Table S4†). The small *p*-values (less than 0.05) and large *F*-value prove the fitness of the model, and its ability to determine the concentration of the three adulterants. These results indicate that there is only a 0.01% chance that an *F*-value this large could occur due to noise. However, the lack of fit (*p*-values more than 0.05) implies that the curvature of the model is not significant relative to the pure error, which could occur due to noise. Non-significant lack of fit is desirable as we want the model to fit, which means that the curvature is not significant. The relatively small difference between the adjusted and predicted *R*^2^ indicates good predictability of the model. Adequate precision measures the signal-to-noise ratio. A ratio greater than 4 is achieved, which indicates an adequate signal and proves that the model can be used to navigate the design space. Residuals *versus* predicted data plots (Fig. S2†) indicated the good fitness of the model as the residuals (errors) are within the specified limits. No significant interaction between the factors was observed. The desirability function selected the best level of each factor, which was then used to optimize the predictive ability of the GA-PLS model. The function suggested using ml, fit% and LV of 10, 82% and 5, respectively (Fig. S3†), with 97.60% desirability.

The suggested ml was applied to GA parameters, while the suggested fit% was used to select the optimum set of wavelengths. The PLS model was constructed using the calibration set and the optimum number of LV (Fig. S1†). The smaller RMSEP of the GA(DoE)-PLS model relative to that of the GA-PLS model suggests better predictive ability of the former model (Fig. 2).

### 3.9 Optimization of ANN model

**Fig. 3:**
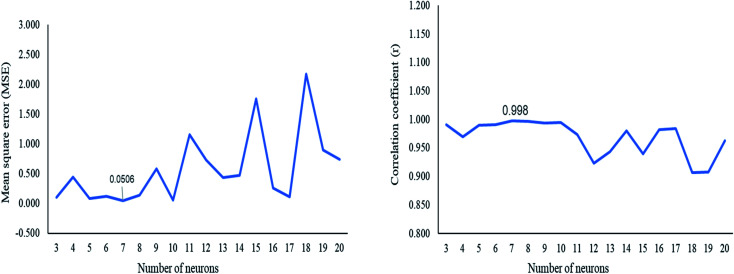
Plot of the mean square error (MSE) and the correlation coefficient (*r*) against the number of neurons in the architecture of GA(DoE)-ANN.

The ANN model was constructed using the GA (DoE) dataset. Different numbers of hidden neurons were tested to select the optimum number of neurons that improved the ANN predictive ability. The optimum number of hidden neurons was seven, which gave a small MSE and correlation coefficient *r* near unity (Fig. 3). The ANN architecture indicated the different layers used for the prediction of the concentration of the three adulterants (Fig. S4†). Purelin–Purelin transfer function was suitable owing to the linear absorbances–concentrations relationship. The TRAINLM-Levenberg–Marquardt backpropagation was preferred as a training function. The GA(DoE)-ANN model was performed on the validation set, and RMSEP was calculated.

### 3.10 Model reproducibility and data prediction

The four models successfully predicted the concentration of the three analytes in the calibration and validation sets (Table 1). Reproducible and precise results (% RSD) were obtained for repeatability and intermediate precision, indicating the success of the four models in analyzing the three adulterants (Table 1). The GA(DoE)-PLS and GA(DoE)-ANN models achieved the best accuracy (low RMSEP) and precision (low % RSD) compared to the other models (Fig. S5 and S6†), owing to the useful GA variable selection tool, the effective DoE optimization approach, and the powerful ANN artificial intelligence (Fig. 2).

### 3.11 Validation of the models

Few methods were reported for the assay of MY in turmeric powder. However, no method was reported for the simultaneous assay of the three adulterants in turmeric powder. Accordingly, we validated the developed chemometric models as per the ICH guidelines.^61^ This was achieved through spiking of the pure turmeric powder with known concentrations of the three adulterants, and the ability of the proposed models to recover the concentration of the spiked adulterants was then assessed. The recovery percentages and the relative standard deviation percentages were calculated (Table 2).

**Table 2:** Determination of metanil yellow, acid orange 7 and lead chromate in spiked turmeric samples

Models	Metanil yellow (recovery% ± RSD%)^a^	Acid orange 7 (recovery% ± RSD%)^a^	Lead chromate (recovery% ± RSD%)^a^
Partial least square	98.67 ± 1.876	100.07 ± 1.840	100.28 ± 1.650
Genetic algorithm-partial least square	99.00 ± 1.746	100.33 ± 1.685	99.65 ± 0.923
Optimized genetic algorithm-partial least square	99.42 ± 1.338	99.28 ± 0.976	100.05 ± 0.477
Artificial neural network using optimized genetic algorithm	99.75 ± 0.938	99.93 ± 0.577	99.97 ± 0.200

a Average of three determinations.

## 4. Conclusion

Four multivariate chemometric models (PLS, GA-PLS, GA (DoE)-PLS and GA(DoE)-ANN) successfully quantified three toxic turmeric adulterants (MY, AO and LCM) in turmeric powder samples to determine the type and amount of the adulterant. The work demonstrated the augmented influence of the GA and the experimental design on the predictive ability of the PLS and ANN models. The four chemometric methods were validated as per the ICH guidelines. The developed methods proved to be fast, cheap and involved minimal sample preparation. The methods can be used for fast surveillance of turmeric powder adulteration in quality control laboratories before including the powder in food recipes to guarantee authenticity, safety and quality.

## Author contributions

All authors contributed to the conceptualization of the work, analysis, investigation and visualization of the data.

## Funding

This research did not receive any specific grant from funding agencies in the public, commercial or not-for-profit sectors.

## Conflicts of interest

There are no conflicts to declare.

## Supplementary Material

RA-012-D2RA00697A-s001
