# Supplementary material for: Experimentally designed chemometric models for the assay of toxic adulterants in turmeric powder
Source: RSC Adv. 2022 Mar 23;12(15):9087–94. doi: 10.1039/d2ra00697a (PMC8985183; doi:10.1039/d2ra00697a)
Supplement: RA-012-D2RA00697A-s001 [file RA-012-D2RA00697A-s001.pdf]

Electronic Supplementary Material (ESI) for RSC Advances.
This journal is © The Royal Society of Chemistry 2022

Supplementary Materials

Supplementary Table S1. Configuration of the genetic algorithms parameters.

Parameter	Value
Population size	20
Maximum generations	50
Mutation rate	0.005
The number of variables in a window (window width)	2
Percent of the population the same at convergence	50
% Wavelengths used at the initiation	50
Crossover type	Single
Maximum number of latent variables	3
Cross-validation	Random
Number of subsets to divide data into for cross-validation	4
Number of iterations for cross-validation at each generation	2

Supplementary Table S2. Optimized parameters of the genetic algorithms.

Parameters	Level code		
	-1	0	+1
ml	3	8	13
fit%	50%	70%	90%
LV	4	5	6

Supplementary Table S3. Optimized parameters of the artificial neural network.

Parameters	GA(DoE)-ANN [a]		
	Metanil yellow	Acid orange 7	Lead chromate
Hidden neurons number	3-20		
Transfer functions	Purelin–Purelin		
Training function	TRAINLIM		
Learning coefficient	0.001		
Learning coefficient decrease	0.001		
Learning coefficient increase	100		

[a] Artificial neural network using the optimized genetic algorithm

Supplementary Table S4. ANOVA results of the optimized genetic algorithm-partial least square model.

	Metanil yellow			Acid orange 7			Lead chromate		
	F-value	p-value	Conclusion	F-value	p-value	Conclusion	F-value	p-value	Conclusion
Model	100.87	< 0.0001	Significant	21.49	< 0.0001	Significant	85.67	< 0.0001	Significant
Lack of fit	1.96	0.2975	Not significant	7.34	0.0638	Not significant	3.00	0.1981	Not significant
Adjusted R^2		0.9836			0.7321			0.9186	
Predicted R^2		0.8885			0.5801			0.8697	
Adequate Precision		25.0838			8.2623			16.5482	

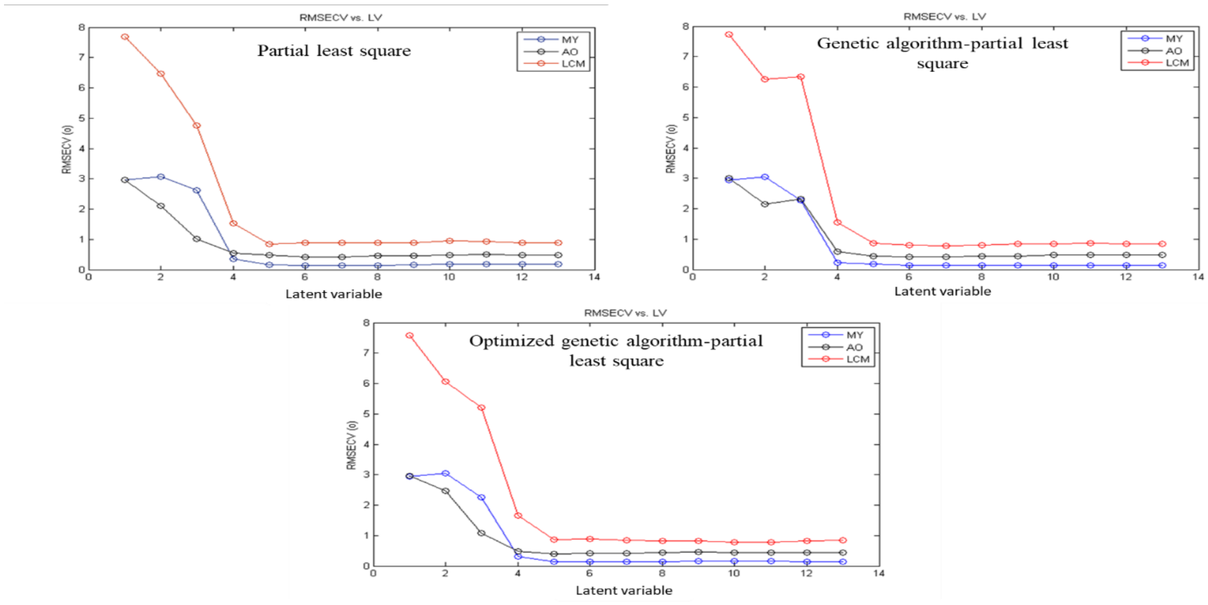

Supplementary Figure S1. RMSECV plot of the cross-validation results of the calibration set as a function of the number of principal components used to construct the proposed models.

*MY: metanil yellow, AO: acid orange 7, LCM: lead chromate.

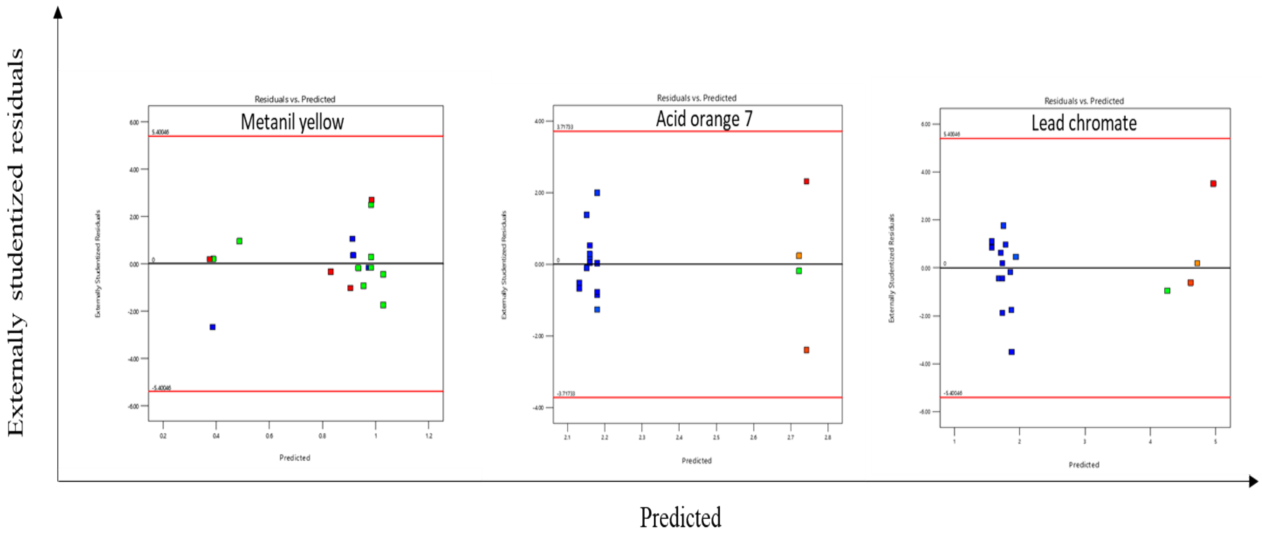

Supplementary Figure S2. Residuals versus predicted data of the standard deviation of the concentration of metanil yellow, acid orange 7 and lead chromate in the optimized genetic algorithm-partial least square (GA(DoE)-PLS) model.

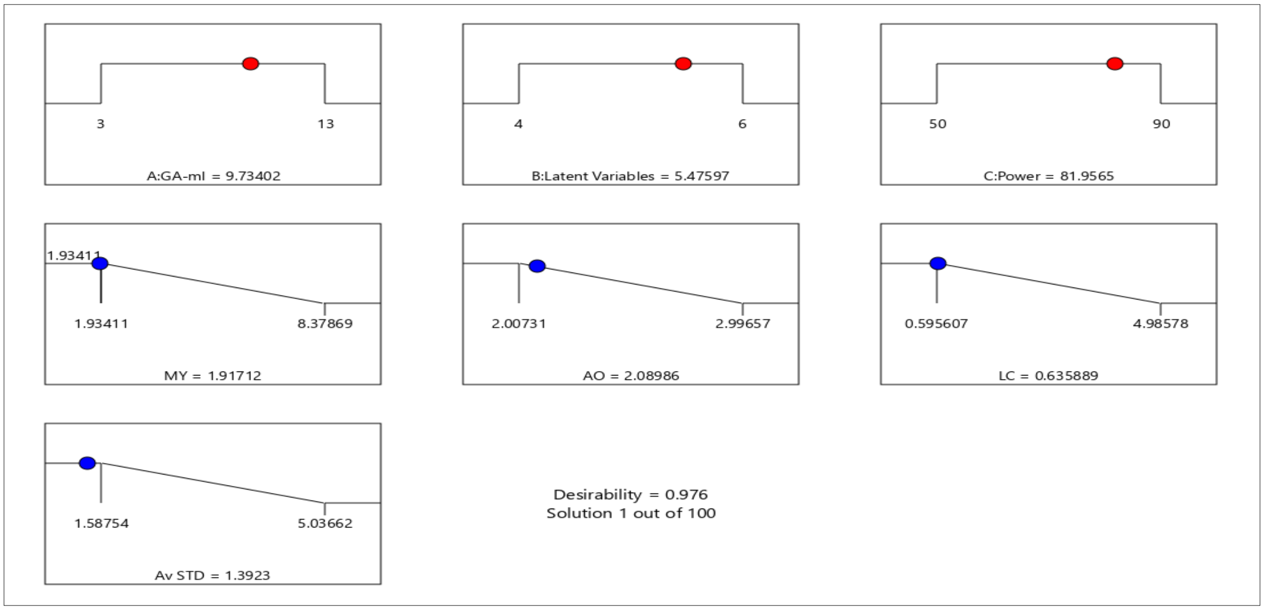

Supplementary Figure S3. Numerical optimization of the three adulterants in optimized genetic algorithm-partial least square (GA(DoE)-PLS) model.

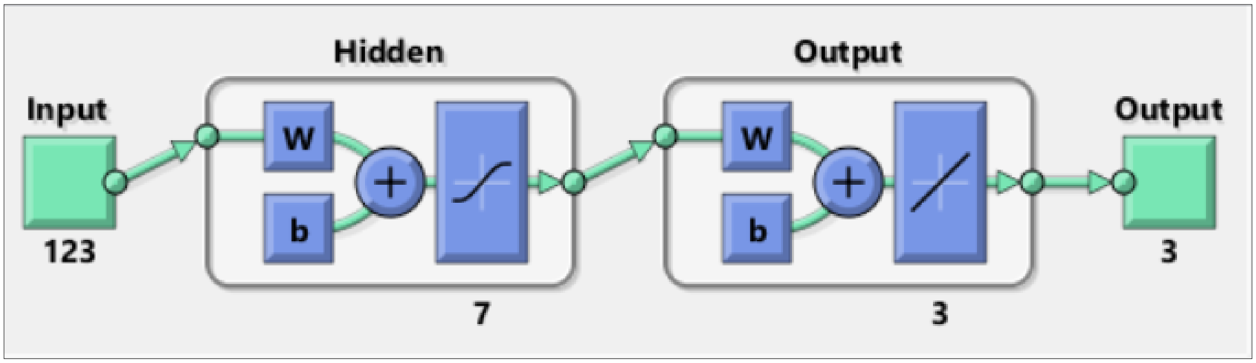

Supplementary Figure S4. Artificial neural network architecture uses different layers for the prediction of the concentrations of the three adulterants.

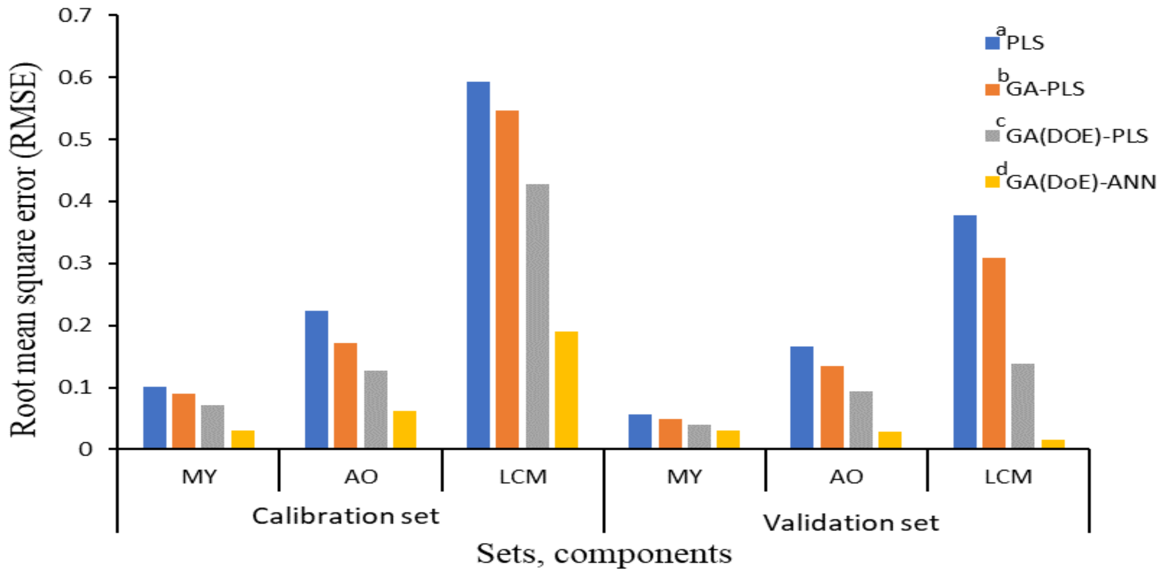

Supplementary Figure S5. Root mean square error of calibration and validation sets for the three adulterants using the four chemometric models.

[a] Partial least square, [b] Genetic algorithm-partial least square, [c] Optimized genetic algorithm-partial least square,

[d] Artificial neural network using optimized genetic algorithm, MY: metanil yellow, AO: acid orange 7, LCM: lead chromate.

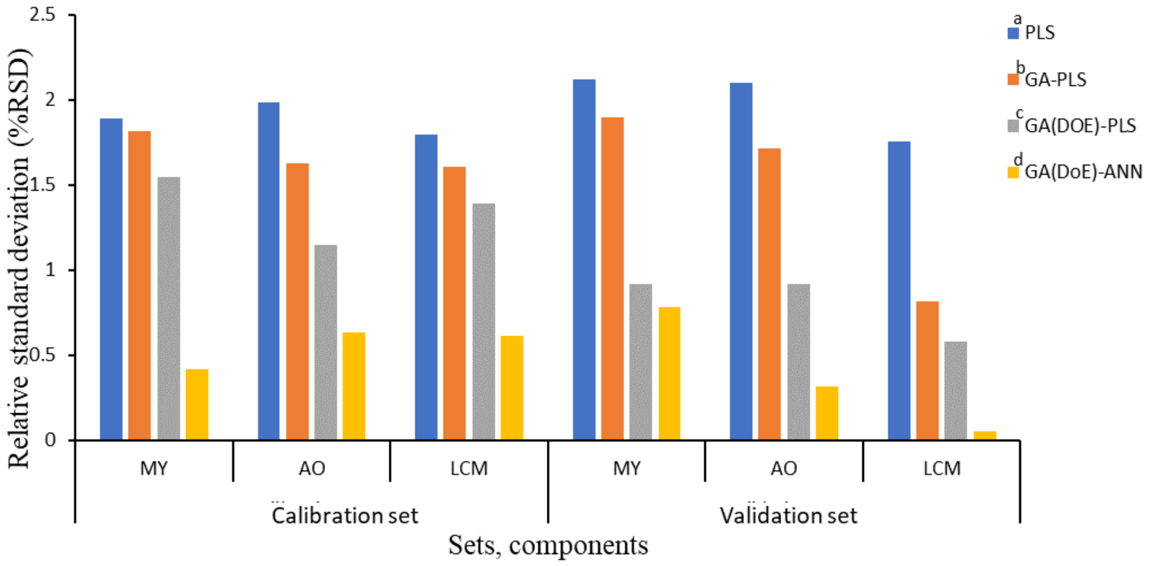

Supplementary Figure S6. The relative standard deviation of calibration and validation sets for the three adulterants using the four chemometric models.

[a] Partial least square, [b] Genetic algorithm-partial least square, [c] Optimized genetic algorithm-partial least square, [d] Artificial neural network using optimized genetic algorithm, MY: metanil yellow, AO: acid orange 7, LCM: lead chromate.
